# The Role of Vitamin D Supplementation Before Coronary Artery Bypass Grafting in Preventing Postoperative Atrial Fibrillation in Patients With Vitamin D Deficiency or Insufficiency: A Systematic Review and Meta-Analysis

**DOI:** 10.7759/cureus.36496

**Published:** 2023-03-21

**Authors:** Saad Ali Ansari, Jasninder Singh S Dhaliwal, Yusra Ansari, Sudeshna Ghosh, Tahir Muhammad Abdullah Khan

**Affiliations:** 1 Internal Medicine, University of California Riverside School of Medicine, Riverside, USA; 2 Internal Medicine, University of Kentucky College of Medicine, Bowling Green, USA; 3 Pulmonary and Critical Care Medicine, University of Kentucky College of Medicine, Bowling Green, USA

**Keywords:** coronary artery bypass grafting (cabg), arrhythmia, post-operative atrial fibrillation, post operative complication, vitamin d supplementation

## Abstract

This study aims to evaluate the role of preoperative vitamin D supplementation before coronary artery bypass grafting (CABG) surgery in preventing postoperative atrial fibrillation (POAF) in vitamin D deficient or insufficient patients. Three randomized controlled trials (RCTs) comprising 448 subjects were selected after a detailed search was conducted on PubMed, Cochrane CENTRAL, Scopus, and Embase in December 2022. Analysis was run using RevMan (version 5.4.1; Copenhagen: The Nordic Cochrane Centre, The Cochrane Collaboration, 2014). The analysis collected risk ratio (RR) and 95% confidence interval (CI) data from the relevant studies, which were then pooled using a random effects model. A significance level of less than 0.05 (p<0.05) was considered significant. Our analysis showed that compared with the standard of care, preoperative vitamin D supplementation in vitamin D deficient and insufficient patients effectively reduced POAF after CABG surgery (RR=0.6, 95% CI=0.4-0.9, P=0.01). There was no significant difference in the duration of hospitalization between the vitamin D supplementation group compared with the control following CABG (mean difference -0.85, 95% CI -2.13 to 0.43, P = 0.19). This meta-analysis shows that preoperative vitamin D supplementation in vitamin D deficient and insufficient patients undergoing CABG can reduce the rate of POAF. As POAF is associated with many complications, providing vitamin D supplementation to individuals with a vitamin D deficiency undergoing CABG can improve long-term cardiovascular outcomes following surgery.

## Introduction and background

Atrial fibrillation (AF) is a common cardiac arrhythmia characterized by irregular and often rapid heartbeat originating in the atria of the heart. Coronary artery bypass grafting (CABG) is a surgical procedure to improve blood flow to the myocardium by bypassing blocked or narrowed coronary arteries. However, CABG can result in postoperative AF (POAF), affecting approximately 20-40% of patients, making it the most common arrhythmia post-CABG. POAF causes adverse outcomes in the postoperative period, increasing the risk of stroke and hemodynamic instability, leading to a prolonged hospital stay and rising healthcare costs. The onset of POAF usually occurs within the first week following surgery, peaking at 2-4 days post-surgery [1]. According to a large study, the cumulative risk of stroke at 10 years in patients with POAF was nearly twice as high as in patients without POAF. Therefore, healthcare providers need to be cautious of this potential complication and take steps to monitor and manage patients who develop atrial fibrillation after CABG [2].

The exact mechanism of post-CABG AF is not fully understood, but it is thought to be related to factors such as inflammation, ischemia, and autonomic nervous system dysfunction [3]. One risk factor for POAF may be low vitamin D levels. Vitamin D is believed to play a role in producing specific cytokines that reduce inflammation and is also a negative regulator of the renin-angiotensin-aldosterone system (RAAS). Theoretically, low vitamin D levels can cause increased inflammation and upregulate RAAS, increasing the risk of POAF [4]. However, extensive studies have not confirmed a definitive association between vitamin D levels and post-CABG AF. Most studies have been observational, with some randomized controlled trials (RCTs) also conducted. Still, their results have been mixed in establishing a correlation between low vitamin D levels and post-CABG AF [5-10]. To examine the impact of vitamin D supplementation on reducing POAF in patients with vitamin D deficiency or insufficiency, we performed a meta-analysis by combining data from three randomized controlled trials. Although the individual trials had a small number of participants, pooling the data provided greater statistical power and a more robust association could be demonstrated.

In this review, we will analyze three RCTs in which vitamin D supplementation was given to patients with low vitamin D levels before undergoing CABG to reduce the incidence of POAF. By investigating the potential impact of vitamin D supplementation, we can identify a new approach to reducing the risk of POAF and improving patient outcomes following CABG.

## Review

Methods

This meta-analysis was conducted according to the preferred reporting items for systematic reviews and meta-analyses (PRISMA) guidelines. First, a thorough search was conducted on PubMed, Cochrane CENTRAL, Scopus, and Embase in December 2022 using the following search terms: (Coronary artery bypass grafting OR CABG) AND (Vitamin D level OR Vitamin D deficiency OR Vitamin D insufficiency OR Vitamin D supplementation) AND (Postoperative atrial fibrillation). A deeper search was then conducted to identify the randomized controlled studies and trials from the articles.

Study Selection

To select the studies, we established the following inclusion criteria: (a) the study must be a published randomized controlled trial, (b) the participants must be adult males or females with vitamin D deficiency or insufficiency who underwent CABG and received either vitamin D supplementation or no supplementation preoperatively, (c) vitamin D deficiency was defined as a vitamin D level less than 20 ng/ml, and vitamin D insufficiency was a vitamin D level between 20 to 30 ng/ml, d) the study should compare the incidence of POAF between the vitamin D supplementation and no vitamin D supplementation groups and (e) we excluded case reports, review articles, and single-arm observational studies, from our search results.

Data Extraction 

Two independent reviewers evaluated the papers based on their title and abstract, and only those that met the above criteria were selected. Subsequently, the entire article was meticulously examined to ensure its eligibility. 

Statistical Analysis

The statistical analysis of this article was performed using RevMan (version 5.4.1; Copenhagen: The Nordic Cochrane Centre, The Cochrane Collaboration, 2014). The analysis collected risk ratio (RR) and 95% confidence interval (CI) data from the relevant studies, which were then pooled using a random effects model. A statistical level of less than 0.05 (p<0.05) was considered significant. A Chi-square test was conducted to assess the differences between groups, and the results were visually displayed using a forest plot. We also evaluated heterogeneity across all studies using Higgins I2, where a value of less than 50% was deemed significant.

Results

Literature Search Results

**Figure 1:**
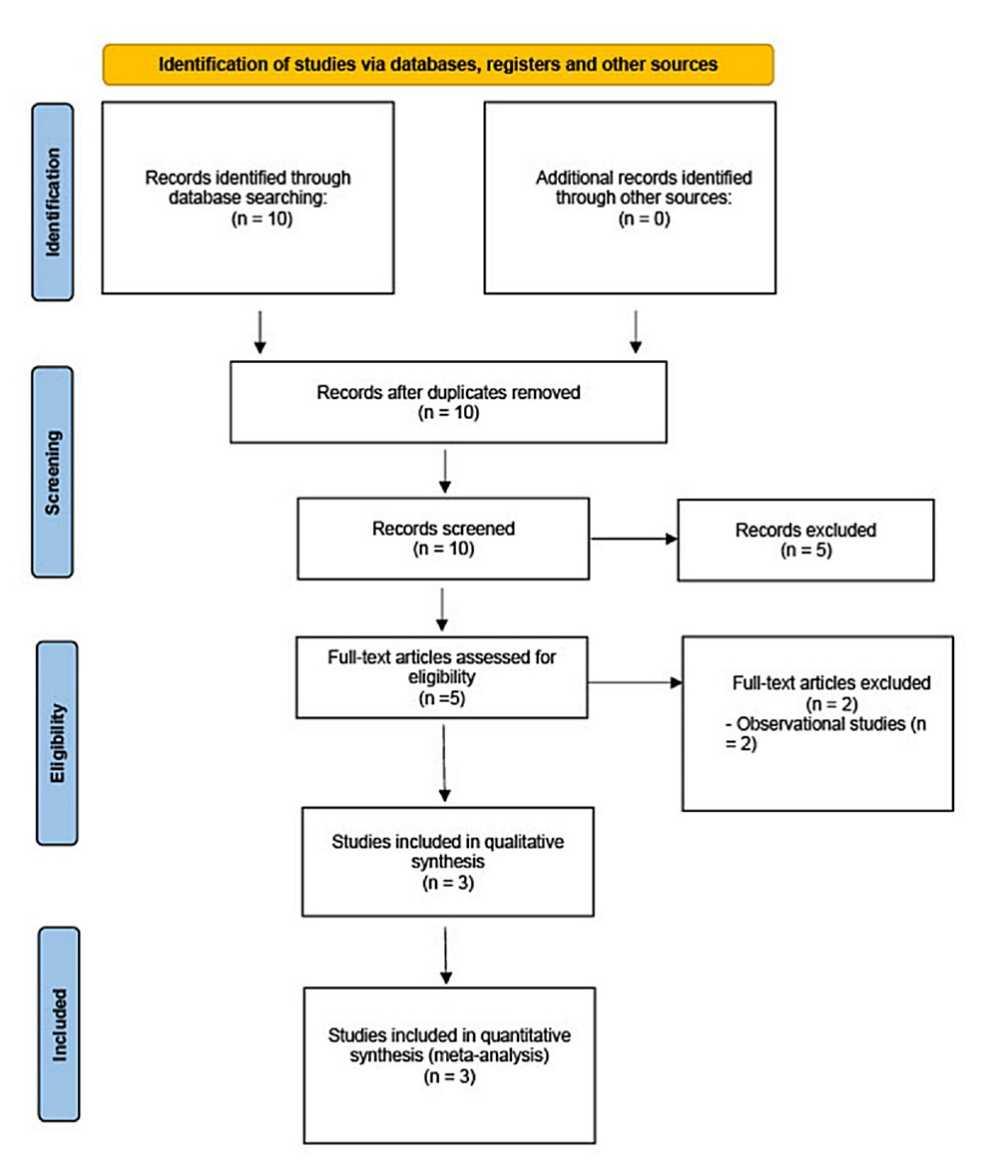
PRISMA flowchart for selection of studies PRISMA: Preferred Reporting Items for Systematic Reviews and Meta-analyses

After a comprehensive search across all databases, 10 studies were identified as potential candidates. Upon individual examination of each study, only three of them were found to meet the inclusion criteria. A PRISMA flow chart (Figure 1) has been provided to summarize the literature search results.

Study Characteristics

The studies that met the inclusion criteria involved 448 patients with vitamin D deficiency or insufficiency who underwent CABG. Among them, 219 received vitamin D supplementation in the preceding five days to 48 hours before surgery, while 229 did not. A summary of the baseline characteristics of vitamin D supplementation and control groups in all the studies can be found in Tables 1-2.

**Table 1:** Baseline characteristics across vitamin D supplementation group. SD: standard deviation, BMI: body mass index, COPD: chronic obstructive pulmonary disease, LVEF: Left ventricular ejection fraction, ACE-i/ARB: angiotensin-converting enzyme inhibitor/angiotensin receptor blocker. * across both vitamin D deficiency and insufficiency groups.

Study name	Ceritt et al. [8]	Hakan et al. [9]	Talasaz et al. [10]
Year	2018	2019	2022
Design	RCT	RCT	RCT
Country	Cyprus	Turkey	Iran
Vitamin D supplementation group (n)	68	58	93
Age (mean ± SD)	63.8 ± 9.3	64.9 ± 10.5	59.3 ± 10.1
Male (%)	53.7	27.6	75.3
Female (%)	46.3	72.4	24.7
BMI (kg/m2), (mean ± SD)	Not given	26.74 ± 3.96	28.04 ± 6.43
Diabetes Mellitus (%)	37.9	29.31	46.2
Hypertension (%)	62.5	62.07	50.5
Hyperlipidemia (%)	32.8	Not given	52.6
Smoking history (%)	24.6	Not given	26.8
COPD (%)	28.7	Not given	3.2
Duration of hospitalization (Day ± SD)	8.1 ± 2.3	7.14 ± 1.97	7.40 ± 2.75
LVEF (%)	62.7 ± 6.9	60.68 ± 8.5	43.2 ± 10.2
Left atrial diameter (mm)	38.9	38.2 ± 4.9	39.53 ± 3.47
Beta-Blocker therapy (%)	86	36.21	80.6
Statin therapy (%)	92	46.5	87.1
ACE-i/ARB therapy (%)	72	39.65	49.4
Vitamin D level (deficiency group) (ng/ml)	11.4 ± 4.9	10.77 ± 3.21	14.43 ± 12.13​​​​​ *
Vitamin D level (insufficiency group) (ng/ml)	24.6 ± 3.7	25.13 ± 3.45	14.43 ± 12.13​​​​​ *
Serum Creatinine (mg/dl)	1.03 ± 0.29	0.85 ± 0.17	1.00 ± 0.26

**Table 2:** Baseline characteristics across the control group. SD: standard deviation, BMI: body mass index, COPD: chronic obstructive pulmonary disease, LVEF: Left ventricular ejection fraction, ACE-i/ARB: angiotensin-converting enzyme inhibitor/angiotensin receptor blocker. * across both vitamin D deficiency and insufficiency groups.

Study name	Ceritt et al. [8]	Hakan et al. [9]	Talasaz et al. [10]
Year	2018	2019	2022
Design	RCT	RCT	RCT
Country	Cyprus	Turkey	Iran
Control group (n)	68	58	103
Age (mean ± SD)	62.7 ± 8.9	65.21 ± 9.98	62.22 ± 9.12
Male (%)	51.9	22.41	71.8
Female (%)	48.1	77.59	28.2
BMI (kg/m2), (mean ± SD)	Not given	26.62 ± 2.71	27.63 ± 5.64
Diabetes Mellitus (%)	35.4	37.93	58.2
Hypertension (%)	63.7	58.62	56.3
Hyperlipidemia (%)	34.7	Not given	52.4
Smoking history (%)	27.1	Not given	28.1
COPD (%)	26.9	Not given	3.88
Duration of hospitalization (Day ± SD)	7.9 ± 2.1	7.81 ± 2.25	9.58 ± 4.0
LVEF (%)	61.3 ± 7.1	63.81 ± 10.16	43.21 ± 10.9
Left atrial diameter (mm)	39.3	37.45 ± 4.18	39.96 ± 4.1
Beta-Blocker therapy (%)	89	44.83	86.4
Statin therapy (%)	94	53.45	88.3
ACE-i/ARB therapy (%)	70	29.31	59.2
Vitamin D level (deficiency group) (ng/ml)	10.9 ± 5.2	11.91 ± 3.88	14.94 ± 13.87​​​​​ *
Vitamin D level (insufficiency group) (ng/ml)	24.9 ± 3.9	27.56 ± 0.70	14.94 ± 13.87​​​​​ *
Serum Creatinine (mg/dl)	1.01 ± 0.23	0.85 ± 0.21	1.00 ± 0.29

Results of Meta-Analysis

**Figure 2:**
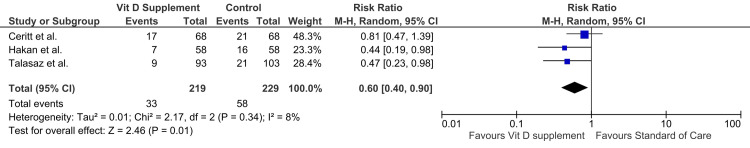
Forest plot comparing vitamin D supplementation with no vitamin D supplementation in preventing postoperative atrial fibrillation in vitamin D deficient or insufficient patients undergoing coronary artery bypass grafting. Source: References [8-10]

Incidence of postoperative atrial fibrillation: Out of the 219 patients with vitamin D deficiency or insufficiency who were administered vitamin D supplementation, 33 developed POAF (15.06%). In contrast, among the 229 patients in the control group who did not receive vitamin D supplementation, 58 (25.32%) developed POAF. Our study's results demonstrated that preoperative vitamin D supplementation in vitamin D deficient or insufficient patients who underwent CABG reduced the incidence of POAF (RR=0.60, 95% CI=0.40-0.90, P=0.01). Figure 2 depicts the forest plot for these findings.

**Figure 3:**
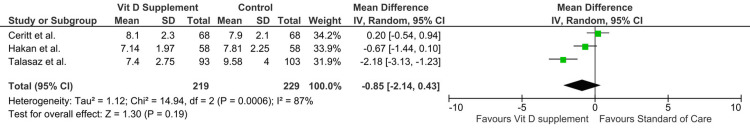
Forest plot of mean difference in length of hospitalization between vitamin D supplementation group compared with control. Source: References [8-10]

Length of Hospitalization: There was no significant difference in the duration of hospitalization between the vitamin D supplementation group compared with the control following CABG (Mean difference -0.85, 95% CI -2.13 to 0.43, P = 0.19). The forest plot for these findings is given in Figure 3.

Discussion

Various complications are associated with coronary artery bypass grafting during the postoperative period. These complications may include respiratory failure requiring extended ventilatory support, congestive heart failure, and postoperative infections such as pneumonia, urinary tract infections, and superficial and deep wound infections. Additionally, there is a risk of renal failure, pericarditis, myocarditis, mediastinitis, cerebrovascular accidents, arrhythmias, etc. Among these complications, POAF is one of the most common and can lead to several adverse early and late outcomes [11].

The etiology of POAF is not fully understood. However, it is believed that various factors may contribute to its development, including systemic and local inflammation from surgical trauma and pericardial disruption with the production of pro-inflammatory cytokines and oxidative stress from ischemia and reperfusion during the use of cardiopulmonary bypass. In addition, electrolyte disturbances in the postoperative period can also play a role in the pathogenesis of postoperative atrial fibrillation. Some of the risk factors for postoperative atrial fibrillation include advanced age, male gender, white race, and the presence of comorbidities such as chronic obstructive pulmonary disease, congestive heart failure, hypertension, prior history of atrial fibrillation, diabetes mellitus, hyperthyroidism, chronic kidney disease, obesity, and echocardiographic evidence of left atrial enlargement and left ventricular dysfunction [3,12].

POAF is linked with unfavorable outcomes in the postoperative phase, including a heightened risk of stroke, extended hospitalization, and increased healthcare expenses. In addition, patients who experience persistent postoperative atrial fibrillation typically require long-term anticoagulation, which is connected to bleeding complications. Given these potential consequences, it is crucial to identify modifiable risk factors that can be addressed to decrease the incidence of postoperative atrial fibrillation [1].

While it has been suggested that vitamin D deficiency may be a risk factor for postoperative atrial fibrillation as it plays a role in reducing inflammation and acts as a negative regulator of RAAS and in improving overall cardiovascular health, some of the prior studies failed to demonstrate a significant correlation between low levels of vitamin D and the occurrence of new or postoperative atrial fibrillation conclusively [3,4]. Our review analyzed three randomized controlled studies in which people with vitamin D deficiency were given vitamin D supplementation in the preoperative period. The results of our meta-analysis suggest that vitamin D supplementation in vitamin D deficient patients in the preoperative period can reduce the incidence of postoperative atrial fibrillation. We also examined the length of stay in the vitamin D supplementation group. Prolonged hospital stays are linked to higher hospital costs and may increase the likelihood of acquiring hospital-acquired infections. Although there was a trend towards a reduced length of stay in the vitamin D supplementation group, the difference was not statistically significant, likely due to the small number of patients across the three trials. Conducting additional randomized trials may provide further insights into the potential reduction in the length of stay associated with vitamin D supplementation.

While interpreting our meta-analysis, it's essential to consider certain limitations. Firstly, our study included only three trials, comprising 448 patients. Further randomized controlled trials are required to understand the significance of vitamin D supplementation in lowering the incidence and complications associated with POAF. Secondly, the trials we included did not conduct a stratified analysis of patients based on their comorbidities, which may introduce potential confounding factors that could not be entirely ruled out. Thirdly, as all the trials were conducted in countries in the Middle East and Mediterranean region, the generalizability of our meta-analysis results may be limited due to a lack of variation in racial characteristics and demographics.

As POAF can lead to unfavorable outcomes, our meta-analysis suggests that providing vitamin D supplementation to individuals with a vitamin D deficiency undergoing CABG can decrease the incidence of POAF and enhance long-term cardiovascular outcomes following CABG. Therefore, vitamin D levels of patients undergoing CABG should be routinely checked, and those with vitamin D deficiency or insufficiency should be given preoperative vitamin D supplementation.

## Conclusions

Postoperative atrial fibrillation after coronary artery bypass surgery is a common complication linked to poorer outcomes during the recovery period. One possible contributing factor to the development of postoperative atrial fibrillation is a deficiency in vitamin D. However, supplementing with vitamin D can potentially be beneficial in reducing the occurrence of postoperative atrial fibrillation for patients with low levels of this vitamin. Further randomized controlled trials may be needed to further study the impact of vitamin D supplementation in preventing both postoperative and non-postoperative atrial fibrillation across patient populations.

## References

[1] Dobrev D, Aguilar M, Heijman J, Guichard JB, Nattel S (2019). Postoperative atrial fibrillation: mechanisms, manifestations and management. Nat Rev Cardiol.

[2] Benedetto U, Gaudino MF, Dimagli A (2020). Postoperative atrial fibrillation and long-term risk of stroke after isolated coronary artery bypass graft surgery. Circulation.

[3] Lopes LA, Agrawal DK (2022). Post-operative atrial fibrillation: current treatments and etiologies for a persistent surgical complication. J Surg Res (Houst).

[4] Thompson J, Nitiahpapand R, Bhatti P, Kourliouros A (2015). Vitamin D deficiency and atrial fibrillation. Int J Cardiol.

[5] Ohlrogge AH, Brederecke J, Ojeda FM (2022). The relationship between vitamin D and postoperative atrial fibrillation: a prospective cohort study. Front Nutr.

[6] Emren SV, Aldemir M, Ada F (2016). Does deficiency of vitamin D increase new onset atrial fibrillation after coronary artery bypass grafting surgery?. Heart Surg Forum.

[7] Özsin KK, Sanrı US, Toktaş F, Kahraman N, Yavuz Ş (2018). Effect of plasma level of vitamin D on postoperative atrial fibrillation in patients undergoing isolated coronary artery bypass grafting. Braz J Cardiovasc Surg.

[8] Cerit L, Özcem B, Cerit Z, Duygu H (2018). Preventive effect of preoperative vitamin D supplementation on postoperative atrial fibrillation. Braz J Cardiovasc Surg.

[9] Kara H, Yasim A (2020). Effects of high-dose vitamin D supplementation on the occurrence of post-operative atrial fibrillation after coronary artery bypass grafting: randomized controlled trial. Gen Thorac Cardiovasc Surg.

[10] Talasaz AH, Salehiomran A, Heidary Z, Gholami K, Aryannejad H, Jalali A, Daei M (2022). The effects of vitamin D supplementation on postoperative atrial fibrillation after coronary artery bypass grafting in patients with vitamin D deficiency. J Card Surg.

[11] Montrief T, Koyfman A, Long B (2018). Coronary artery bypass graft surgery complications: a review for emergency clinicians. Am J Emerg Med.

[12] Greenberg JW, Lancaster TS, Schuessler RB, Melby SJ (2017). Postoperative atrial fibrillation following cardiac surgery: a persistent complication. Eur J Cardiothorac Surg.

